# Role of PLZF as a tumor suppressor in prostate cancer

**DOI:** 10.18632/oncotarget.19813

**Published:** 2017-08-02

**Authors:** Yang Jin, Hatice Zeynep Nenseth, Fahri Saatcioglu

**Affiliations:** ^1^ Institute for Cancer Genetics and Informatics, Oslo University Hospital, Oslo, Norway; ^2^ Department of Biosciences, University of Oslo, Oslo, Norway

**Keywords:** promyelocytic leukemia zinc finger, prostate cancer, tumor suppressor, androgens

## Abstract

The promyelocytic leukemia zinc finger (PLZF), also known as ZBTB16 (Zinc Finger And BTB Domain Containing 16), is a transcription factor involved in the regulation of diverse biological processes, including cell proliferation, differentiation, organ development, stem cell maintenance and innate immune cell development. A number of recent studies have now implicated PLZF in cancer progression as a tumor suppressor. However, in certain cancer types, PLZF may function as an oncoprotein. Here, we summarize our current knowledge on the role of PLZF in various cancer types, in particular prostate cancer, including its deregulation, genomic alterations and potential functions in prostate cancer progression.

## INTRODUCTION

The promyelocytic leukemia zinc finger protein (PLZF), also known as ZBTB16 (Zinc Finger and bric à brac, tramtrack, and broad (BTB) Domain Containing 16) or ZFP145 (Zinc Finger Protein 145), is a member of the Krüppel C2H2-type zinc-finger protein family and encodes a zinc finger transcription factor. PLZF was first identified in an acute promyelocytic leukemia (APL) patient with a t(11;17) reciprocal chromosomal translocation which resulted in an in-frame fusion with the retinoic acid receptor alpha (RARA) gene. The PLZF-RAR fusion protein suppresses normal functions of PLZF and RARA, and is thus implicated in APL development. Since then significant new findings established PLZF as a multifaceted protein that regulates diverse biological processes, including cell proliferation, apoptosis, cell cycle, differentiation, and development (for a review, see [1]). Consistently, decreased PLZF expression has been associated with several types of cancer [2], in particular prostate cancer (PCa), which is the main focus of this review.

### Physiological and cellular functions of PLZF

PLZF has a wide tissue distribution. Early studies focused on PLZF primarily in stem cells and early progenitor cells [3], but it is now established that PLZF is expressed in many cell types [4]. PLZF expression is higher in the brain, lung, endocrine and male-specific tissues including prostate and testis compared to other tissues (http://www.proteinatlas.org/) [4]. PLZF is predominantly nuclear in these tissues, except for the lung, in which PLZF expression is primarily cytoplasmic [5]. In the prostate, PLZF expression is largely restricted to the luminal cells, with expression weak/absent in basal or stromal cells [5, 6].

Previous studies showed that under normal physiological conditions PLZF is essential for normal differentiation and development. In 2000, a Plzf knockout mouse model was developed that displayed abnormal patterning of all limb skeletal structures [7]. Plzf^−/−^ mouse has major musculoskeletal-limb defects with homeotic transformations of vertebral segments, deformed cartilage and skeletal patterning, and alterations in digit formation. The abnormal skeletal phenotype resembles the general features of luxoid mouse, which was first described in the 1950s [8, 9]. Genomic analysis revealed that the luxoid phenotype results from a frameshift mutation in the Plzf gene, which causes inhibition of progenitor cell apoptosis in the autopod region, thus blocking further limb development, possibly due to inhibition of Hox gene expression [9]. There are defects in germ cell production in both luxoid mutants and Plzf^−/−^ mice [9, 10], indicating a crucial role for Plzf in spermatogonial self-renewal. In germline progenitor cells, Plzf acts as a repressor of c-kit receptor expression to prevent spermatogenesis; in addition, it acts as an activator of the mTORC1 inhibitor Regulated in Development and DNA damage response 1 (REDD1) to prevent stem cell depletion [11].

PLZF has also been associated with stem and progenitor cell maintenance in hematopoietic stem cells (HSC) and neural progenitors. HSCs from luxoid mice exhibit an amplified aging phenotype associated with loss of stemness [12], while forced expression of PLZF immortalizes HSCs and myeloid progenitors [13]. PLZF is broadly expressed in earlier neural progenitors. Elevated PLZF expression sustains cells in a progenitor state and restricts neural differentiation by increasing FGFR3 expression and STAT3 activity [14]. PLZF maintains self-renewal of stem cells in a cell-type specific manner and promotes stem cell differentiation, such as osteogenesis and chondrogenesis of mesenchymal stem cells. Recent findings implicated PLZF to play a critical role in the development of essentially all of the innate-like features of invariant Natural Killer T (NKT) cells [15–17].

**Figure 1:**
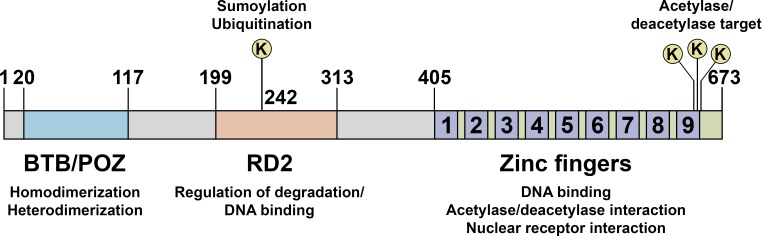
Schematic representation of PLZF functional domains PLZF protein consists of a bric à brac, tramtrack, and broad/poxvirus and zinc finger (BTB/POZ) domain, a second repression domain (RD2), and a zinc finger domain. The BTB/POZ domain allows PLZF to form homodimers as well as heterodimeric complexes with co-repressor molecules, such as N-CoR, SMRT and Sin3A. Lysine 242 in RD2 is a sumoylation and ubiquitination site. Sumoylation is critical for the transcriptional repression activity of PLZF whereas ubiquitination is involved in its degradation. The zinc finger domain, composed of nine Krüppel-like C2H2 zinc fingers, is responsible for DNA binding on target gene promoters. Acetylation of lysine residues at the zinc finger domain enhances DNA binding and transcriptional repression by PLZF.

PLZF mediates its effects mainly by modulating transcription, acting either as an activator or a repressor. PLZF consists of a BTB/POZ domain, a second repression domain (RD2), and a zinc finger domain (Figure 1). The BTB/POZ domain allows PLZF to form homodimers as well as heteromeric repressive complexes with NCoR, SMRT and Sin3A [18, 19]. The zinc finger domain, composed of nine Krüppel-like C2H2 zinc fingers, is responsible for DNA binding on target gene promoters [7]. PLZF activity is also regulated by post-translational modifications, such as acetylation [20–22], ubiquitination [23] and sumoylation [24] (Figure 1).

The mechanisms of PLZF-mediated repression of gene expression and the genes affected were recently reviewed in detail [1]. Briefly, PLZF recruits histone deacetylases (HDACs) and promotes the formation of the SMRT-mSin3-HDAC-NCoR corepressor complex to inhibit transcription [18, 19]. In addition, PLZF can repress transcription by directly sequestering transcription factors. For example, PLZF binds the NF-κB p50 subunit and HDAC3 that assemble on the promoters of NF-κB target genes as transcriptionally inactive complexes [21, 22]. This limits active NF-κB p50-p65 heterodimer formation, inhibits target gene expression, and thus leads to suppression of inflammatory responses [21, 22]. PLZF can also induce or suppress gene expression by directly binding to promoters of target genes through its Krüppel-like C2H2 zinc fingers [7]. So far, only a few genes have been shown to be direct PLZF targets [1]. As a BTB/POZ domain containing protein, PLZF can also bind with Cullin 3 (CUL3) to form the E3 ubiquitin ligase complex, which occurs in the cytosol. For example, in Hela cells, PLZF-CUL3-ROC1 complex mediates the ubiquitination of ATG14L and thereby inhibits autophagy [25]. Thus PLZF has both nuclear and cytosolic functions in the cell.

### PLZF and cancer

In early studies, PLZF was primarily linked to haematological malignancies, but it is now also implicated in various solid tumors (Table 1), including melanoma [26, 27], hepatocellular carcinoma [28], pancreatic cancer [29], colon cancer [30, 31], lung cancer [32], thyroid carcinoma [33], glioma [34] and PCa [5, 6, 35, 36].

**Table 1:** Alteration of PLZF in cancer

Tumor type	Alterations	Ref.
Clear cell renal cell carcinoma	Increased expression in tumor compared with normal tissue	[34]
Colon cancer	Increased expression in colorectal cancer cells compared with normal colonocytes	[31, 50]
Glioblastoma	Increased expression in tumor compared with normal tissue	[34]
Hepatocellular carcinoma	Decreased expression in tumor tissue compared to adjacent normal tissue	[28]
Lung cancer	Decreased expression in high grade and high risk lung cancers	[32, 38]
Melanoma	Decreased expression in high risk melanomas compared to low risk melanomas	[26]
Pancreatic cancer	Down-regulation or loss of expression in tumor tissue compared to adjacent normal tissue; aberrant promoter methylation	[29]
Prostate cancer	Down-regulation or loss of expression in high grade tumors; homozygous deletion in CRPC	[5, 6, 35, 36]
Testicular seminoma	Increased expression in tumor compared with normal tissue	[34]
Thyroid carcinoma	Decreased nuclear expression and increased cytosolic expression in papillary thyroid carcinoma tissue compared with normal thyroid tissue	[33]

PLZF expression is high in epidermal melanocytes from the foreskin, but is decreased in melanoma cell lines [26] and its expression is substantially lower in high-risk melanomas compared to low-risk ones [27]. Retroviral transduction of PLZF expression in melanoma cells resulted in a more differentiated, less malignant phenotype both *in vitro* and *in vivo* [26] which appears to be mediated by inhibition of the Pre-B-Cell Leukemia Homeobox 1 (PBX1) and Homeobox B7 (HOXB7) complex [37].

In human hepatocellular carcinoma (HCC), PLZF expression is significantly decreased at both mRNA and protein levels compared with paired adjacent normal tissue [28]. In addition, PLZF expression and several clinical features are positively correlated in HCC patients, such as the alkaline phosphatase levels and prothrombin time [28].

PLZF expression is reduced in pancreatic cancer relative to normal tissue, and is lost in 38 of 247 (15.4%) cases [29]. Loss of, or reduction of, PLZF expression may be due to aberrant PLZF promoter methylation which was detected in 35.2% of pancreatic cancer cases (24).

PLZF expression is elevated in various colorectal cancer cell lines compared with isolated normal colonocytes [30]. It is inversely correlated to and transcriptionally suppressed by the expression of the CCAAT-Displacement-Protein (CUX1) [30]. Interestingly, a novel N-terminally truncated form of PLZF, and not the full length PLZF, is expressed in colorectal cancer cells [31]. The truncated PLZF lacks the N-terminal BTB/POZ domain and thus cannot translocate to the nucleus to inhibit transcription. In contrast to the tumor suppressive function of the intact PLZF protein, the truncated isoform appears to have a role in colorectal cancer cell adhesion and survival [31].

There is moderate to strong expression of PLZF in luminal cells of benign respiratory bronchus and well-differentiated lung adenocarcinoma, but lower levels in the basal layer [5]. Furthermore, loss of PLZF expression is strongly correlated with increased grade, stage, lymph node metastasis and poor survival of lung squamous carcinoma and lung adenocarcinoma [5]. In pulmonary neuroendocrine tumors PLZF expression is negatively correlated to the tumor grade [38]. As in pancreatic cancer, loss of PLZF expression in non-small cell lung cancer may be due to promoter hypermethylation [39]. Interestingly, PLZF mainly localizes to the nucleus in the low-grade lung neuroendocrine tumor [40], while it is predominantly localized to the cytosol in the normal lung and lung adenocarcinoma [32].

PLZF is expressed in the normal thyroid and various thyroid lesions. Interestingly, whereas in the normal thyroid, adenomatous lesions, and follicular adenoma PLZF is mainly in the nucleus, in papillary thyroid carcinoma and anaplastic thyroid carcinoma it is primarily expressed in the cytosol (33). Furthermore, increased cytosolic PLZF expression was correlated with capsular invasion and lymph node metastasis in papillary thyroid carcinoma [33]. The basis for these intriguing findings on differential PLZF intracellular localization and correlation to thyroid pathologies is currently unknown.

The studies summarized above suggest that the full-length nuclear PLZF may function as a tumor suppressor protein. On the other hand, there is evidence suggesting that in some cases PLZF may also serve as an oncoprotein. For example, IHC analysis showed a significant increase in the percentage of nuclear PLZF-positive cells in clear cell renal cell carcinoma, glioblastoma, and testicular seminoma tissues, compared with the corresponding normal tissues [34]. In addition, PLZF knockdown in clear cell renal cell carcinoma Caki-1 cells inhibited xenografted tumor growth in BALB/c-nu mice [34]. These data suggest that under certain circumstances PLZF may function as an oncoprotein.

It should be noted that many of the studies on PLZF so far utilized individual cell culture models *in vitro*, in particular while studying specific molecular interactions. Since the structural organization of living tissue composed of many cell types interacting with one another may affect these processes, further studies should focus on novel *in vitro* models, such as organoids and explant systems, but also *in vivo* models, e.g. utilizing the Plzf^-/-^ mice in the genetic background relevant to the cancer of interest.

### PLZF in prostate cancer

#### Androgen regulation of PLZF expression

Androgen signaling is one of the most central pathways in PCa [41]. This is the basis for androgen deprivation therapies (ADT), including chemical/surgical castration, a mainstay treatment for metastatic PCa in the clinic. Although ADT is initially effective, most PCa patients become resistant and eventually develop castration resistant PCa (CRPC). PLZF expression was increased in castrated rat ventral prostate after androgen replacement [42]. This was further validated in androgen receptor (AR) positive PCa cell lines LNCaP [6, 42], VCaP and 22Rv1 [35]. In addition, the antiandrogen bicalutamide inhibited PLZF expression in LNCaP cells [35]. Consistent with this, PLZF expression is undetectable in the AR deficient PCa cell lines DU145 and PC3 and ectopic expression of AR restored PLZF expression in DU145 cells [43], confirming androgen regulation of its expression, which is rapid and transient. These findings establish that PLZF is an androgen activated gene.

PLZF expression is also regulated by PTEN/AKT/FOXO3 signaling, another important pathway in PCa cells [36]. There are seven putative consensus forkhead response element (FHRE) binding sites within the PLZF flanking sequences, including the 2.3 kb segment upstream of the transcription start site and the first exon. One site (FHRE1, +18∼+24) that localizes within the first exon is essential for FOXO3 binding to the PLZF promoter [36]. FOXO3 knockdown in LNCaP cells inhibits PLZF expression while ectopic expression of constitutively active FOXO3 in PC3 PCa cells induces it. Consistently, PLZF, PTEN and FOXO3 protein expression are positively correlated in a cohort of human PCa specimens [36].

### PLZF in prostate cancer progression

IHC analysis of multiple PCa cohorts indicated no significant change in PLZF expression in benign versus malignant prostate; furthermore, there was no correlation to Gleason score or T-stage [5]. Another IHC study found strong expression of PLZF in both benign prostate glands and low grade prostate tumors (Gleason pattern 3), but no significant difference between them [36]. However, in high grade PCa (Gleason pattern 4 and 5), PLZF expression was reduced/lost in 26% of the tumors and in up to 86% in metastatic specimens [5]. Another study also reported a decrease in PLZF expression in high grade PCa compared with low grade PCa [36]. In addition, PLZF expression was significantly lower in human bone metastatic CRPC tumor samples compared with hormone sensitive primary PCa tumors [35]. Whole-exome sequencing of metastatic CRPC specimens revealed that 5–7% of tumors harbor PLZF homozygous deletions [35]. These findings suggest that PLZF may have a role in PCa progression.

#### Tumor suppressive functions of PLZF in prostate cancer

Several studies have assessed the possible role of PLZF in PCa. For example, ectopic expression of PLZF inhibits proliferation of PCa cell lines LNCaP [42], DU145 [43], and PC3 [36]. Conversely, inhibition of PLZF expression by siRNA or shRNA in LNCaP cells results in outgrowth [6, 35]. Recent data suggest that PLZF knockdown promotes androgen-independent growth in LNCaP cells and it leads to resistance to the antiandrogen enzalutamide (32). Consistent with this, PLZF re-expression reverses androgen-independent growth (32). In addition, PLZF expression inhibited tumor formation by 22Rv1 cells (a CRPC model) under castrate levels of circulating androgens [35]. These results are consistent with the notion that PLZF is involved in CRPC development.

#### Molecular mechanisms of PLZF action

The data published so far suggest that PLZF acts as a tumor suppressor primarily via its ability to inhibit transcription. For example, the PLZF-RARα fusion protein that arises in APL potently silences retinoic acid-regulated gene expression. Similarly, engineered PLZF fusion with estrogen receptor-α (ERα) or AR leads to inhibition of their target gene expression [44, 45]. In addition, PLZF can directly interact with retinoic acid receptor (RAR), ERα, glucocorticoid receptor (GR) and vitamin D receptor (VDR) and inhibit their transcriptional activity [46].

In LNCaP cells, PLZF knockdown significantly increases expression of AR target genes, whereas ectopic expression of PLZF suppresses androgen-regulated gene expression and inhibits AR transcriptional activity [6]. This appears to be due to inhibitory direct physical interactions between PLZF and AR in PCa cells, which result in suppression of AR transcriptional activity and inhibition of androgen dependent PCa cell growth [6].

**Figure 2:**
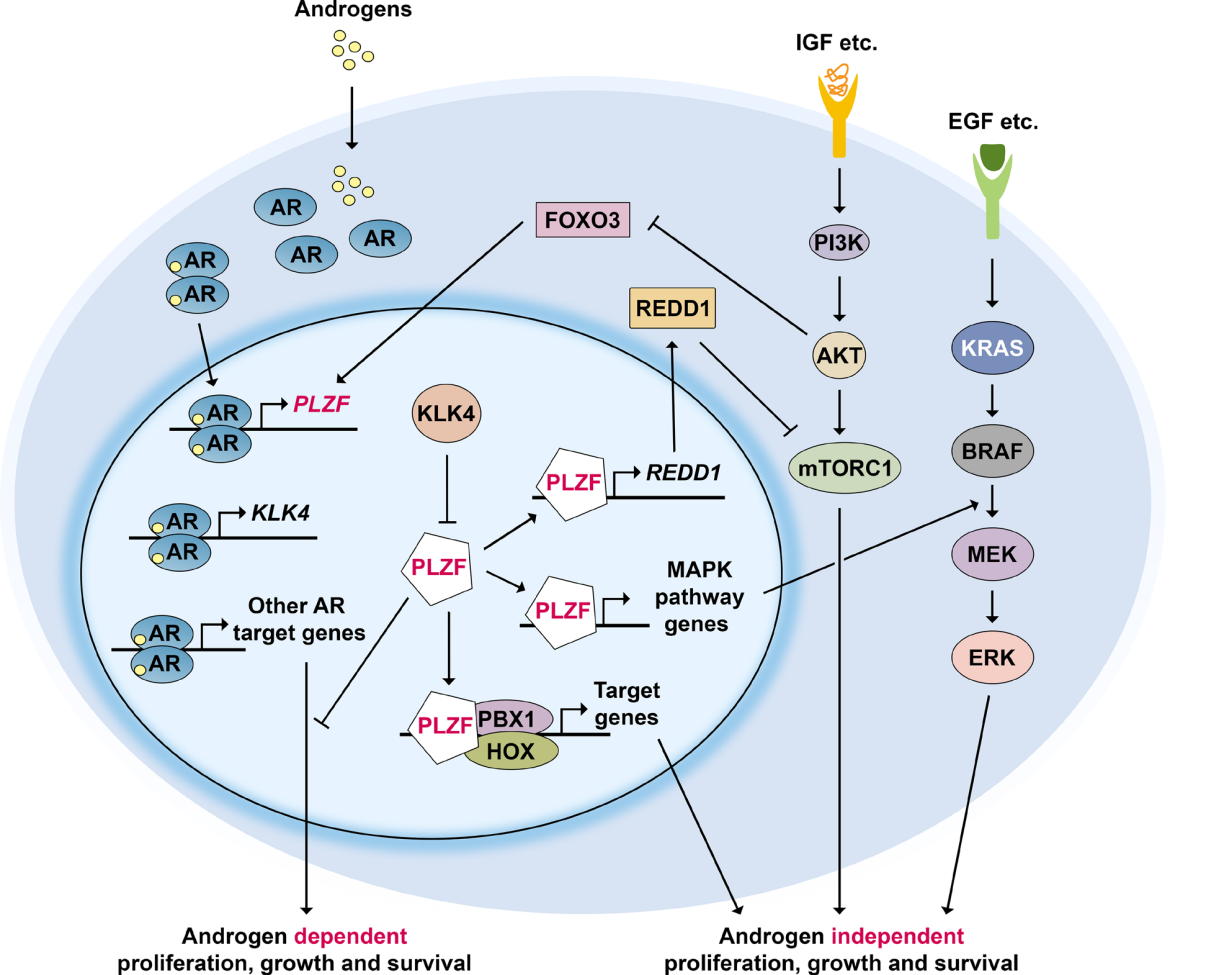
PLZF regulatory network in prostate cancer Androgen receptor (AR) activation induces PLZF expression, while PLZF acts as a negative feedback regulator of AR. In addition, PLZF represses mTORC1 signaling by upregulating REDD1 expression and RAS/RAF/MEK signaling through increasing related gene expression. PLZF can also prevent the formation of PBX1-HOX heterodimers. Activation of PI3K-AKT pathway inhibits FOXO3 which normally activates PLZF expression. Highly prostate enriched protein KLK4 whose expression is androgen dependent, is a key regulator of PLZF: it physically interacts with and inhibits PLZF inhibited pathways, resulting in tumor growth.

As summarized above, PLZF expression in PCa cells is rapidly induced by androgens, while PLZF acts as a negative feedback regulator of AR. Interestingly, this feedback loop is inhibited by another androgen induced protein, Kallikrein Related Peptidase 4 (KLK4), whose expression is highly prostate enriched [6]. KLK4 expression is significantly increased in human PCa and blocking its expression inhibits proliferation and increases apoptosis [6]. Furthermore, siRNA targeting of KLK4 by systemic nanoliposomal delivery results in dramatic regression of xenografted PCa tumors in nude mice, establishing KLK4 as a key regulatory switch for PCa growth [6]. Mechanistic studies identified PLZF as an interacting partner for KLK4 that inhibits PLZF stability [6]. This relieves the inhibitory action of PLZF on androgen signaling in PCa cells resulting in tumor growth [6] (Figure 2).

Another pathway that is critical for proliferation and survival of PCa cells is PI3K-AKT-mTOR signaling [47, 48]. A negative regulator of this pathway, the tumor suppressor protein PTEN, is mutated in the majority of PCa during progression. Earlier work in mouse germ-line progenitor cells has shown that PLZF induced expression of the mTOR inhibitor REDD1 [11]. PLZF is also required for REDD1 expression in PCa cells [6]. Consistent with this, chromatin immunoprecipitation analysis showed that PLZF bound to two distinct sites upstream of the human REDD1 promoter in an androgen dependent fashion [6] (see model in Figure 2). In summary, PLZF activates REDD1 expression and inhibits mTOR activation; KLK4 expression blocks PLZF-mediated REDD1 expression that results in activation of mTOR signaling, giving rise to tumor growth [6].

Global gene expression analysis in PCa cells revealed that PLZF-repressed genes are significantly enriched in the MAPK signaling pathway, including five genes with PLZF-binding sites in their regulatory regions: RRAS, MKNK2, DDIT3, JUND, and JUN (31). In keeping with these data, PLZF knockdown induces phospho-ERK1/2 expression upon EGF stimulation in LNCaP cells [35] (Figure 2). In addition, PLZF inhibits expression of the homeodomain transcription factor PBX1 in androgen independent PCa cells [43]. Loss of PLZF releases PBX1 repression and may allow formation of a heterodimer with HOX, another homeodomain transcription factor. The PBX1-HOX protein complex may then in turn promote androgen-independent cell growth. These pathways and their known and putative interactions are schematically summarized in Figure 2.

### Summary and future perspectives

PLZF is implicated in various cancer types as a tumor suppressor protein. In PCa, there is diminished or loss of PLZF expression in high grade tumors, especially in CRPC. PLZF inhibits PCa cell growth through inhibitory effects on AR, mTOR, and MAPK signaling (Figure 2). Thus, loss of PLZF leads to PCa cell growth, which is supported by *in vitro* and *in vivo* data. PLZF loss may also be involved in progression to CRPC. Therefore, reinstating PLZF expression/reactivation may be a novel strategy for PCa therapy. This could potentially be achieved by gene therapy of wild type PLZF, for example by adenovirus or oncolytic virus delivery, or by small molecules that interfere with inhibitory interactions, similar to that achieved for p53 [49]. In addition, given its interactions with several key signaling pathways in PCa cells, such as KLK4, understanding PLZF biology may help develop new biomarkers and therapeutic targets for PCa.
